# Enhancing the Odontogenic Potential of Human Dental Pulp Stem Cells via Platelet-Rich Plasma Exosomes through Modulation of TGF-β1 and Dentin Sialophosphoprotein

**DOI:** 10.1055/s-0045-1811556

**Published:** 2025-09-08

**Authors:** Dini Asrianti Bagio, Anggraini Margono, Indah Julianto, Shalina Ricardo, Sylva Dinie Alinda

**Affiliations:** 1 Department of Conservative Dentistry, Faculty of Dentistry, Universitas Indonesia, Jakarta, Indonesia; 2 Department of Dermatology and Venerology, Faculty of Medicine, Universitas Sebelas Maret, Surakarta, Indonesia; 3 Dermama Biotechnology Laboratory, Surakarta, Indonesia; 4 Doctoral Program, Faculty of Dentistry, Universitas Indonesia, Jakarta, Indonesia

**Keywords:** platelet-rich plasma, exosomes, dental pulp, stem cells, transforming growth factor, dentin sialophosphoprotein

## Abstract

**Objective:**

Although platelet-rich plasma (PRP) has demonstrated considerable regenerative potential in regenerative endodontic treatment, its clinical efficacy may be limited by the rapid degradation of its bioactive components, leading to inconsistent outcomes. To overcome this challenge, the present study explores the use of nano-sized exosomes derived from PRP—a novel designated as PRP exosomes (PRP-Exo)—as a more stable and targeted biomolecular delivery system to promote odontogenic differentiation within the dentin–pulp complex. The primary objective is to investigate the expression of key odontogenic markers, transforming growth factor-β1 (TGF-β1) and Dentin Sialophosphoprotein (DSPP), in human dental pulp stem cells (hDPSCs) following PRP-Exo treatment.

**Materials and Methods:**

hDPSCs used in this study were biologically stored raw cells harvested at P3-P4. The hDPSCs were starved for 24 hours, then isolated and re-cultured using the enzymatic digestion method until reaching 5 × 10 ^4^ cells/well. Then, culture media were supplemented using osteogenic conditioned media (OCM): 10 mM β-glycerophosphate, 50 µg/mL ascorbic acid, and 100 Nm dexamethasone. The hDPSCs were seeded in different treatments in the following groups: (1) the control group: hDPSCs in DMEM (Dulbecco's Modified Eagle Medium) + OCM; (2) the experimental group: hDPSCs in DMEM + OCM + 5% PRP-Exo. The PRP-Exo was isolated using the qEV isolation methods (Izon, Advisains) diluted into 5% PRP-Exo. PRP-Exo was analyzed and characterized using nanoparticle tracking analysis (NTA; ViewSizer 3000, Horiba, Kyoto, Japan), followed by specific exosome surface markers CD63+ and CD81 + ). TGF-β1 and DSPP expression of hDPSCs was quantified using an enzyme-linked immunosorbent assay (ELISA) kit (Cat. EL-H0111, Elabscience, Wuhan, Hubei), following the manufacturer's protocol, on an ELISA microplate reader under a wavelength of 405 nm. A qualitative result was obtained by Alizarin red staining up to 21 days.

**Results:**

From the NTA result, it was shown that PRP-Exo, which was isolated in this study, has a particle size range of 30 to 150 nm, a homogeneous shape of particles, with several particles after dilution (1.2 × 10 ^6^ particles/mL). It was also proven that 99.19% PRP-Exo in this study has a specific protein surface marker of exosome (CD 63 + ; CD81 + ). The highest TGF-β1 and DSPP expression of hDPSCs after culturing in PRP-Exo was observed on day 14th, and it was statistically significant ( *p* < 0.05). The qualitative results of the Alizarin red staining test were also consistent.

**Conclusion:**

The higher expression of TGF-β1 and DSPP and a larger amount of Ca ^2+^ mineral nodule deposition after 21 days of evaluation of hDPSCs after being treated with PRP-Exo proves that PRP-Exo has potential in the odontogenic process of dentinal pulp complex.

## Introduction

The dentinal–pulp complex is an anatomically and physiologically inseparable unit, comprising dentin and pulp tissue. Both dentin and pulp are specialized connective tissues of mesodermal origin, derived from the dental papillae during tooth development. 1 2 Together, they form the pulp–dentin complex, with mineralized dentin representing the mature outcome of cellular differentiation and maturation. Sharing a common embryological origin, dentin and pulp are interconnected both anatomically and physiologically, with odontoblasts playing a crucial role in the dentin–pulp complex. 3 4

Dentin contains several noncollagenous proteins, including dentin phosphoprotein (DPP), dentin matrix protein 1 (DMP1), dentin sialoprotein (DSP), osteopontin, osteocalcin, and bone sialoprotein. Additionally, it houses proteoglycans, small amounts of phospholipids, and various growth factors (GFs), such as bone morphogenic proteins, insulin-like growth factors, and transforming growth factor-β (TGF-β). 5 Notably, dentin sialophosphoprotein (DSPP) plays a key role in regulating dentin mineralization by initiating the formation of mineral crystals and stimulating the regeneration of hard tissue through the induction of intrafibrillar collagen mineralization. 5 6

Dental pulp has a primary function in maintaining tooth homeostasis, regulating the need for oxygen and nutrients that are important for teeth. 7 Pulpal vascularization was only obtained from blood vessels that entered the root canal through the root apical foramen, with a relatively small diameter of the apical foramen. 6 Thus, the anatomical condition of the pulp surrounded by dentin hard tissue creates a low compliance environment for the pulp tissue due to limited expansion during pulpal inflammation, hypoxia of pulp cells (even in normal conditions), and lack of a collateral vascular system. 6 7 8

The dentin–pulp complex forms reparative dentin as a defense against irritations or stimuli that can lead to reversible pulp inflammation, which is formed due to dentin response with odontoblast-like cell formation as a result of differentiation of human dental pulp stem cells (hDPSCs) as progenitor stem cells in dental pulp. 9 10

Conversely, pro-inflammatory mediators, including cytokines and chemokines, play a crucial role in defending against pathogens within dentin tubules; however, their activity may also induce cellular damage and promote the development of a low-compliance pulp environment, which has minimal collateral vascularization, limits both the number of immune cells in the pulp and the number of blood derived GFs that play a role in pulp healing. 11 The occurrence of pulp inflammation can cause an increased release of reactive oxygen and nitrogen species, which inhibit cell migration and proliferation, because of cell mitochondrial dysfunction, which, if it continues, causes disruption of the structure (coding) and function (messenger RNA) of dental pulp DNA and can eventually lead to cell death or apoptosis. 12

Over the years, it was believed that dental pulp has its response to inflammation and can create regenerative processes in the dentin–pulp complex. 2 7 8 hDPSCs, as the progenitor cells in dental pulp, play an important role in this process; thus, the number of these cells is limited. 13 14 Therefore, regenerative procedures in endodontics have been proposed by the American Association of Endodontics in 2013 with the concept of revascularization in open apex dental treatment. 15 However, it has been stated lately that the concept of revascularization is not fully accepted as a concept of tissue regeneration or revitalization of dental pulp, because it was reported in the longitudinal evaluation that most cases of revascularization should still undergo root canal treatment. 15 16

It was revealed by previous research that it is not only stem cells, GFs, and scaffolds that play a role in the concept of regeneration of the pulp–dentin complex tissue, but the niche biology and intercellular communication created in a favorable environment are also important in the regeneration process of the dentin–pulp complex. 17 For this reason, there was a changing paradigm in the concept of cell-free therapy in regenerative endodontics, which aims to utilize endogenous stem cells in root canals to initiate the migration, proliferation, and differentiation processes of hDPSCs through the release of GFs and protein biomolecule signaling pathways that are following niche biology in root canals; this concept was believed to support dental pulp revitalization. 17 18

The use of secretome as microvesicle protein from blood, known as platelet-rich plasma (PRP), has gained increasing popularity with widespread applications in diverse regenerative fields in the last few years, especially in cell-free-based therapy and endodontics. The use of this exogenous blood source was believed to induce a favorable niche biology of dental pulp to be able to maintain its regeneration process. 19 20 21 22 23 The understanding of the systematic scientific rationale for PRP as a biological product is far from complete. Some studies have reported that many PRP-derived biomolecules fail to be protected from the phospholipid membrane, which may be damaged by lytic enzymes from the extracellular environment and lose their biological activity quickly. Other systematic reviews have stated that there needs to be more standardization for PRP preparations, varied PRP classifications, and basic protocols of PRP clinical applications, making some inconsistent views regarding their clinical outcomes and performances. 24 25 26

Therefore, in the past decade, extracellular vesicle (EV)-based subcellular therapies have emerged as more promising than stem cell-based therapy or using secretome in regenerative therapies. 27 28 29 EVs are natural nano-sized membrane vesicles encapsulated by phospholipid bilayers, which can be secreted by various types of cells in normal or stress conditions, which can be subdivided as exosomes (30–150 nm in diameter), microvesicles (50 nm–1 μm in diameter), and apoptotic bodies (100 nm–5 μm in diameter). Exosomes are small vesicles secreted by various cell types, including blood cells. They contain bioactive molecules such as proteins, lipids, and nucleic acids (RNA and DNA fragments). Exosomes act as messengers that recipient cells can take up, transferring their cargo and influencing various cellular processes. Exosomes have been found to play roles in intercellular communication, immune modulation, tissue regeneration, and more. 28 29 30 31 32 33

Although previous studies have demonstrated that PRP-derived exosomes—particularly thrombin-activated platelet-derived exosomes (TaPDEs)—can enhance vascular endothelial growth factor (VEGF) expression and promote the migration of hDPSCs, 34 their specific role in dentin–pulp complex regeneration remains unclear. This study is the first to investigate the odontogenic potential of PRP-derived exosomes (PRP-Exo) by evaluating their ability to upregulate key regenerative markers, such as TGF-β1 and DSPP, in hDPSCs. The null hypothesis of this study states that PRP-Exo has no significant effect on the expression levels of TGF-β1 and DSPP in hDPSCs. By testing this hypothesis, the study aims to determine the potential of PRP-Exo as a novel cell-free therapeutic approach for dentin–pulp complex regeneration in endodontic applications.

## Materials and Methods

This study was approved by the ethical committee of the Faculty of Dentistry, Universitas Indonesia (No. 32/Ethical Approval/FKGUI/VIII/2023; Protocol No. 070530623) with biologically stored raw material from the previous study (No. 82/ethical approval/FKG UI/2019; Protocol No. 070940819)—the informed consent of all the adults who participated in this study was obtained before the study.

### Human Dental Pulp Stem Cell Culture

**Fig. 1:**
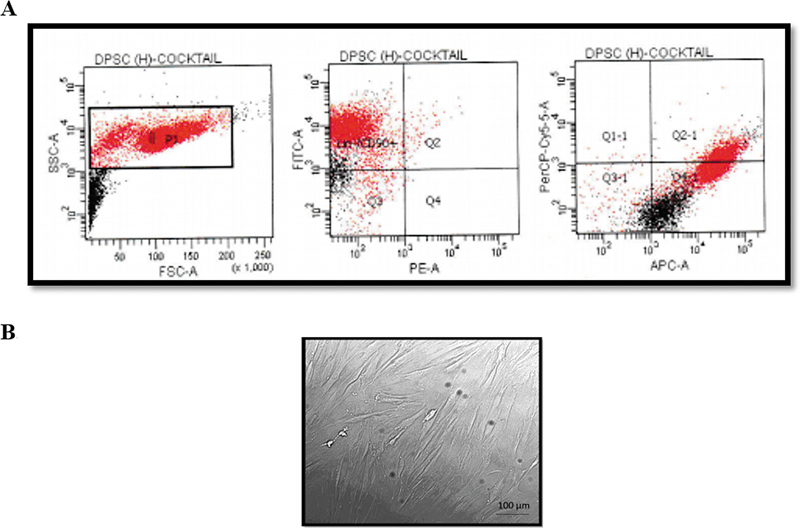
The results of flow cytometry immunophenotyping specific for hDPSCs in this study were as follows: positive cocktail 97.3% for CD90 + ; 96.8% for CD105 + , and 97.7% for CD73+ (>75%). Negative cocktail results: 0.5% for LinNeg (<2%) ( **A** ); qualitative result of hDPSCs after 80% confluency ( **B** ). hDPSCs, human dental pulp stem cells.

hDPSCs used in this study were biologically stored raw cells from the previous study (No. 82/ethical approval/FKGUI/IX/2019; Protocol No. 070940819). The hDPSCs were isolated and re-cultured with an enzymatic digestion method, then harvested at P3-P4, and incubated until reaching 80% confluence. 35 36 The hDPSCs were continued with serum starvation for 24 hours in Dulbecco's Modified Eagle Medium (DMEM; Thermo Fisher Scientific Inc., Massachusetts, United States) supplemented with 1% fetal bovine serum. Then for the next 24 hours, culture media was supplemented using osteogenic culture media (OCM) containing 0.1% β-glycerophosphate, 0.1% ascorbic acid, and 0.1% dexamethasone, then cultured in two 24-well plates (for enzyme-linked immunosorbent assay [ELISA]) and a single 48-well plate (for Alizarin Red test). The hDPSCs were seeded in different treatments in the following groups: (1) the control group: hDPSCs in DMEM + OCM; (2) the experimental group: hDPSCs in OCM + 5% (50 µg/mL) PRP-Exo. All the groups had three biological replicates (Triplo), with two experimental times ( *n* = 2). The hDPSCs were analyzed via flow cytometry mesenchymal stem cell (MSC) analysis using FACSverse (BD Biosciences) with an MSC-positive cocktail (CD90 + , CD105 + , and CD73 + ) and a negative cocktail (LinNeg) for hDPSCs ( Fig. 1A ). The qualitative result of hDPSCs was shown in Fig. 1B .

### Isolation of PRP Exosomes

**Fig. 2:**
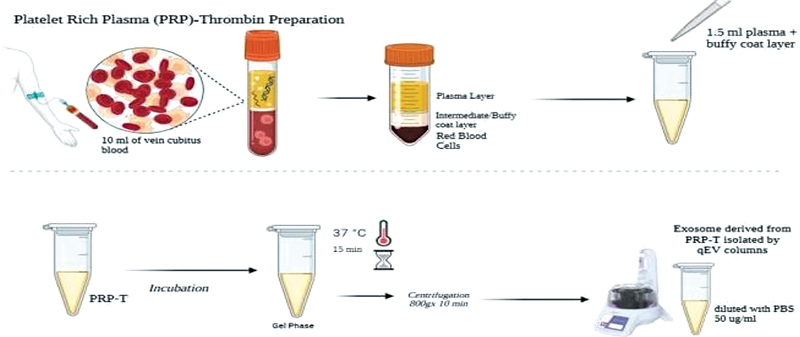
PRP-Exo isolation using qEV isolation methods. PRP-Exo, platelet-rich plasma-exosome.

In total, 10 mL blood was collected from three donors (with informed consent), with normal red blood cell (RBC) count (hemoglobin: 13–15 g/dL; hematocrit: 38–46%; and erythrocytes: 3.8– 5.2 × 10 ^6^ /μL), and donors that matched the following inclusion criteria for this study: age, 12 up to 18 years, healthy without any systemic disease; not taking aspirin; no smoking habit; and no alcohol consumption. After blood was collected, specific centrifugation steps were taken to separate plasma and isolate PRP (should be done at 20–24°C during centrifugation steps). Further centrifuging of PRP was done to concentrate the PRP-thrombin (PRP-T), which was activated after adding 0.5 mL of calcium gluconate (based on a previous study). 37 Then PRP-T was incubated until the gel phase was centrifuged again at 800 g for 10 minutes. The PRP-Exo was isolated using the qEV isolation methods (Izon; Fig. 2 ). The PRP- Exo was then diluted in PBS based on the previous study; 50 µg/mL or 5% PRP-Exo. 34 38 39 40

### PRP-Exo Characterization: Sizes, Morphology, and Immunophenotyping

**Fig. 3:**
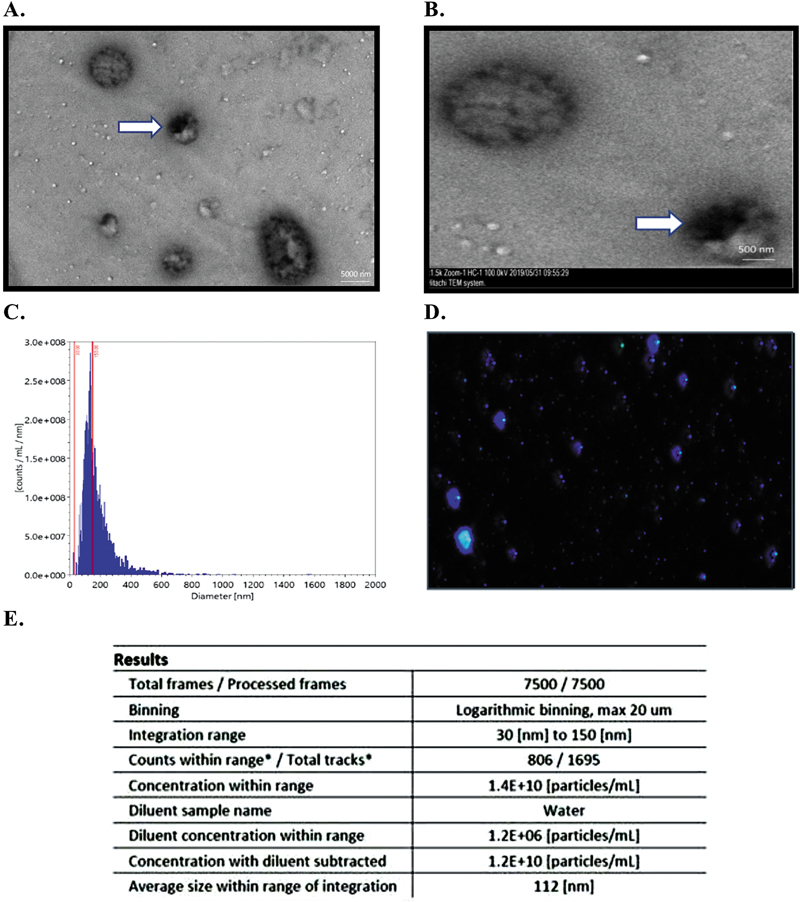
TEM and NTA evaluation of PRP-Exo. A double lipid layer of exosome vesicles was seen at the outer part: vesicles and exosomes by 5,000 nm evaluation (white arrow). From TEM analysis, it was shown that many vesicles contained exosomes by 2,000 nm evaluation ( **A, B** ). NTA graphic results ( **C** ). Fluorescently labeled exosomes visualized using NTA with fluorescence detection mode ( **D** ). NTA analysis result of particles sizes in PRP-Exo ( **E** ). NTA, nanoparticle tracking analysis; PRP-Exo, platelet-rich plasma-exosome; TEM, transmission electron microscopy.

Nanoparticle tracking analysis (NTA; ViewSizer 3000, Horiba, Kyoto, Japan) was employed in this study to analyze and characterize the morphology of exosomes based on their size and concentration. NTA provides detailed information on the size distribution and particle concentration within a sample. 32 As exosomes undergo random Brownian motion in a liquid medium, their trajectories are captured by the instrument's camera. The scattered light from each particle is analyzed to determine its size and velocity, which are influenced by both particle size and the viscosity of the surrounding fluid. The instrument's software tracks the movement of individual exosomes over time to calculate their hydrodynamic diameter. The resulting data are displayed as a histogram, illustrating the size distribution of exosomes within the sample ( Fig. 3A–D ). Additionally, the software calculates the concentration of exosomes per unit volume based on the total number of particles detected ( Fig. 3C and 3E ).

**Fig. 4:**
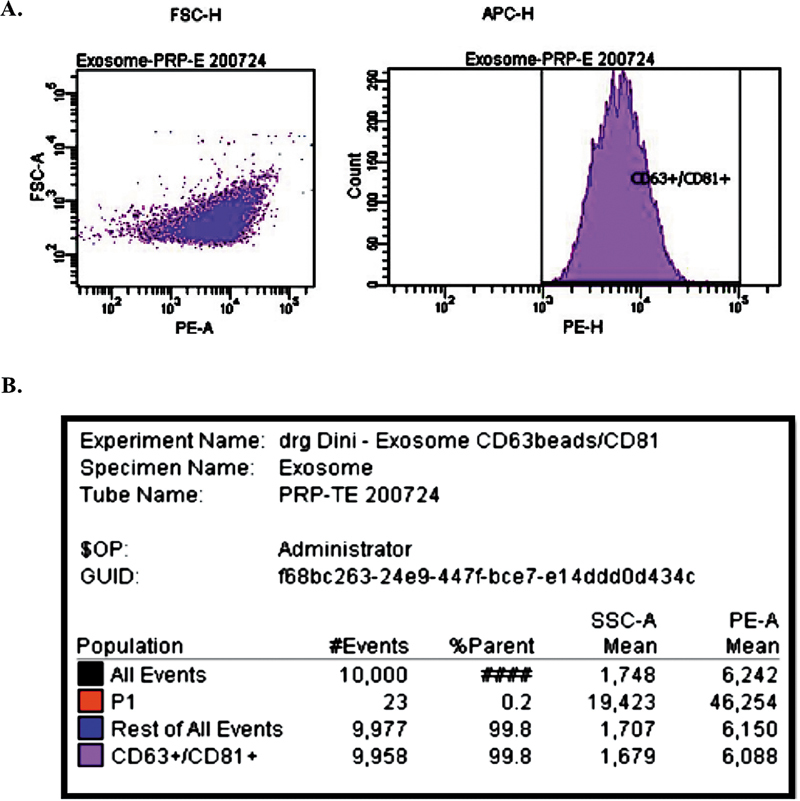
PRP-Exo immunophenotyping result of exosome-specific surface marker test. In graph ( **A** ), the identification of the shape of exosomes formed from blood plasma (P1) is shown, specifically through FSC-H ( *x* -axis) for identifying the size of the exosome protein molecules (30–150 nm) versus SSC-H ( *y* -axis) for identifying the complexity of the exosome protein molecules (FSC-H vs. SSC-H). The histogram graph can express the specific exosome markers CD63 +/CD81 + . Based on the immunophenotyping flow cytometry test of CD63 +/CD81+ exosomes, it can be concluded that 99.8% of the exosome population (P1) expresses CD63 +/CD81+ ( **B** ). PRP-Exo, platelet-rich plasma-exosome.

The physical characteristics of PRP-Exo were analyzed with standard identification methods using an exosome immunophenotyping specific surface marker test (CD63 +/CD81 + ; ab267479 Exosome Isolation and Analysis Kit—Flow Cytometry, Plasma, Abcam, Shanghai, China), following the manufacturer's instructions ( Fig. 4 ).

### Analysis of TGF-β1 and DSPP Expression

The quantitative evaluations of the TGF-β1 and DSPP expression of the hDPSCs using an ELISA 41 kit (Cat. EL-H0111, Elabscience, Wuhan, Hubei), following the manufacturer's protocol, on an ELISA microplate reader under a wavelength of 405 nm. The cells of the control and experimental groups were placed in a 96-well plate ELISA reader. The control and experimental groups' (TGF-β1 and DSPP) expressions were measured after 7 and 14 days of incubation.

### Alizarin Red Staining

Samples of control and PRP-Exo groups were incubated for 14 and 21 days to undergo the Alizarin Red staining test (Sigma-Aldrich, Massachusetts, United States). 41 This test was conducted as a qualitative analysis of the mineralization, Ca ^2+^ deposition nodule formation.

### Statistical Analysis

The TGF-β1 and DSPP expressions of the different groups were compared using a two-way ANOVA test. All the data were analyzed using GraphPad Prism (GraphPad Prism, 10.3.1.509 version, Modern Slavery Act, United Kingdom).

## Result

### hDPSC Culture Cells

Based on the results of stem cell marker testing on pulp stem cells (hDPSCs), it was identified that the cells that were re-cultured from the previous study were pulp stem cells (hDPSCs). The result by flow cytometry immunophenotyping specific for hDPSCs is presented in Fig. 1 .

### PRP-Exo Characterization: Sizes, Morphology, and Immunophenotyping

The PRP-Exo characterization morphology was shown by TEM evaluation ( Fig. 3A, B ), and the standard deviation of 24 nm ± 0.22 and particle modal sizes of PRP-Exo in the range of 112 to 135 nm were shown in NTA graphic results ( Fig. 3C ). Particle size in the total concentration of PRP-Exo is 1.4 × 10 ^10^ particles/mL, and in 5% PRP-Exo is 1.2 × 10 ^6^ particles/mL ( Fig. 3C ). Fluorescently labeled exosomes were visualized using NTA with fluorescence detection mode. This image confirms the presence and distribution of exosomes in the PRP-Exo preparation ( Fig. 3D ; Report # 202203161111004, ViewSizer 3000, Horiba, Kyoto, Japan) (supported data available).

The physical characteristics of PRP-Exo were analyzed with standard identification methods using an exosome immunophenotyping specific surface marker test (CD63 +/CD81 + ). It was shown that the immunophenotyping result of PRP-Exo is 99.9%. This result can confirm that the protein containing PRP-Exo in this study is an exosome ( Fig. 4A, B ).

### TGF-β1 and DSPP Expression of PRP-Exo in hDPSCs

It was demonstrated that the highest TGF-β1 expression in hDPSCs was observed in the PRP-Exo group at 14 days of evaluation (840.57 pg/mL), compared with the control group (18,37 pg/mL), and this difference was statistically significant ( *p* < 0.05). At 14 days, it showed a rapid elevation of TGF-β1 expression; on the contrary, it was decreased in 14 days to the control group.

**Fig. 5:**
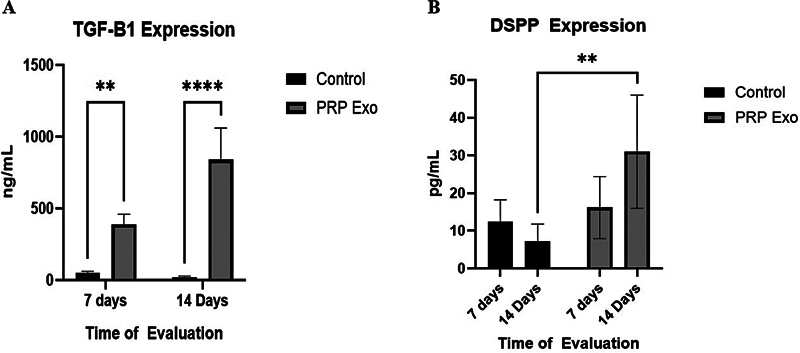
TGF-β1 and DSPP expression of PRP-Exo in hDPSC result of ELISA evaluation. TGF-β1 expression of hDPSCs after culturing in PRP-Exo-conditioned media for 7 to 14 days of evaluations ( **A** ) and DSPP expression of hDPSCs after culturing in PRP-Exo-conditioned media for 7 to 14 days of evaluations ( **B** ). DSPP, dentin sialophosphoprotein; ELISA, enzyme-linked immunosorbent assay; hDPSCs, human dental pulp stem cells; PRP-Exo, platelet-rich plasma-exosome; TGF-β1, transforming growth factor-β1.

In contrast to the TGF-β1 expression, the highest DSPP expression was observed in the PRP-Exo group at 14 days (23.5 ng/mL), compared with the control (6.10 ng/mL), and this difference was statistically significant ( *p* < 0.05; Fig. 5 and Table 1 ). It was shown that even though hDPSCs are cultured in OCM, the DSPP expression will decrease after 14 days (on 7-day evaluation, the expression was similar in control vs. PRP-Exo groups). On the other hand, with supplemented PRP-Exo, the expression of DSPP in the experimental group of PRP-Exo was increased after 14 days ( Fig. 5 and Table 1 ).

**Table 1:** Quantitative result of TGF-β1 and DSPP expression in PRP-Exo and the control groups

Group	Time	Expression (mean ± SD)	
		TGF-β1 (ng/mL)	DSPP (pg/mL)
**C** ontrol	7 days	48.28 ± 11.08	10.40 ± 5.01
	14 days	18.36 ± 9.17	6.10 ± 3.59
**PRP-EXO**	7 days	390.94 ± 26.71	13 ± 7.32
	14 days	840.57 ± 20.66	23.50 ± 15.07

Abbreviations: DSPP, dentin sialophosphoprotein; PRP-Exo, platelet-rich plasma-exosome; SD, standard deviation; TGF-β1, transforming growth factor-β1.

### Alizarin Red Staining Test

Samples of control and PRP-Exo groups were incubated for 14 and 21 days to undergo the Alizarin Red staining test (Sigma-Aldrich, Massachusetts, United States). A qualitative analysis of the mineralization nodule formation, because of the Alizarin red test ( Fig. 5 ), shows that a larger number of nodules are formed by calcium deposition as well as a larger marker area group of nodules on 21 days. This is correlated with the results of previous odontogenic marker expression.

## Discussion

hDPSCs are multipotent cells within the dentin–pulp complex capable of differentiating into odontoblasts, which are responsible for dentin formation. 13 14 Understanding the molecular mechanisms behind this process is critical for advancing regenerative endodontics. TGF-β1 plays a pivotal role in odontogenic differentiation by promoting polarized cell morphology and inducing key markers such as DSPP and DMP-1. DSPP, a major extracellular matrix protein in dentin, is essential for mineralization and structural integrity. Enhancing TGF-β signaling may thus improve strategies for dentin regeneration. 5 25 31 42

PRP, a concentrated source of autologous platelets, promotes tissue repair through its abundance of GFs, cytokines, and regenerative proteins that stimulate cell proliferation and angiogenesis. 24 25 26 27 28 However, clinical outcomes remain inconsistent due to nonstandardized preparation protocols. Recent advances highlight a shift toward PRP-Exo, 34 nanosized vesicles that transport bioactive molecules, including proteins, lipids, and nucleic acids. These exosomes function as intercellular messengers, regulating gene expression, modulating immune responses, and promoting tissue regeneration—primarily through their microRNA (miRNA) activity. 38 39 40 However, it is important to note that while there is promising evidence, more studies are needed to fully elucidate the mechanisms and therapeutic potential of blood-based exosomes in modulating GFs and inducing the regeneration process. There is still limited study in the potential of blood-based exosomes in their performance in tissue regeneration, but it was proven that PRP-Exo has great potential in wound healing and re-epithelialization, also in dental pulp regeneration. 34 40 43

Previous studies have demonstrated the regenerative potential of TaPDEs in dental pulp by enhancing hDPSC viability, migration, and pro-angiogenic activity. 34 The 5% TaPDE group showed superior cell viability and the highest migration rate, along with a marked increase in VEGF-A expression between 24 and 72 hours post-culture. TaPDEs in that study were isolated using the Total Exosome Isolation Kit (Invitrogen) and characterized by transmission electron microscopy and exosome-specific surface markers (CD63 + , CD81 + ; Abcam). 34

In contrast, this study used qEV isolation (Izon; Fig. 2 ) to obtain PRP-Exo, followed by morphological analysis via NTA and immunophenotyping (CD63 + , CD81 + ; Fig. 3C–E ). Although the exosome sizes varied slightly from previous reports (30–150 nm; Fig. 3C ), the qEV method yielded a more homogeneous population with a particle concentration of 1.4 × 10 ^10^ particles/mL for PRP-Exo and 1.2 × 10 ^6^ particles/mL for 5% PRP-Exo (Report # 202203161111004, ViewSizer 3000, Horiba, Kyoto, Japan; Fig. 3D, E ). The high purity (99.8%) confirmed by surface markers aligns with the International Society for Extracellular Vesicles criteria, supporting the use of PRP-Exo as well-defined nano-sized exosomes ( Fig. 4A, B ). 38 39 40 41 42

This study supports previous findings on the pro-angiogenic potential of 5% PRP-Exo and further demonstrates their role in dentin–pulp complex regeneration. 34 Notably, TGF-β1 and DSPP expression significantly increased in hDPSCs cultured with 5% PRP-Exo-supplemented media. The highest TGF-β1 level was observed at day 14 (840.57 pg/mL) compared with the control group (18.37 pg/mL), with a statistically significant difference ( *p* < 0.05; Fig. 5A and Table 1 ). A marked increase in TGF-β1 expression was also evident at days 7 and 14.

These results align with the established role of TGF-β1 in promoting hDPSC differentiation into odontoblast-like cells, characterized by polarized morphology and upregulation of odontogenic markers such as DSPP and DMP-1. TGF-β1 regulates the cell cycle by inhibiting proliferation and initiating differentiation through the suppression of DNA-binding inhibitory proteins. 25 31 The observed increase in TGF-β1 expression from days 3 to 7 suggests a transition phase from cell proliferation to differentiation, consistent with the early stages of odontogenic commitment. 34 43 44

At day 14, DSPP expression was significantly higher in the PRP-Exo group (23.5 ng/mL) compared with the control (6.1 ng/mL; *p* < 0.05; Fig. 5B and Table 1 ). While DSPP levels declined in the control group after day 7, they continued to rise in the PRP-Exo group, indicating sustained odontogenic activity.

Exosomes support dentinogenesis by being internalized into hDPSCs, where they modulate the niche environment and activate MAPK (p38) signaling, promoting DSPP expression and odontogenic differentiation. DSPP is then cleaved into DSP and DPP, the latter initiating hydroxyapatite formation in the dentin matrix. 41 42

**Fig. 6:**
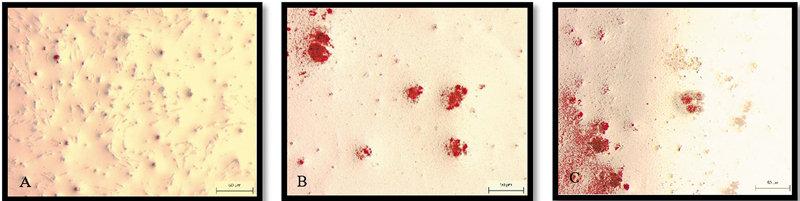
Qualitative result Alizarin Red staining of hDPSCs in the control group ( **A** ); PRP-Exo group after 14 days ( **B** ); and after 21 days ( **C** ). hDPSCs, human dental pulp stem cells; PRP-Exo, platelet-rich plasma-exosome.

This is also in line with the qualitative result of the Alizarin Red staining test, which showed that a larger number of nodules are formed by calcium deposition, as well as a larger marker area group of nodules in 21 days ( Fig. 6 ). Based on this finding, the null hypothesis is rejected.

Nevertheless, the study has certain limitations, particularly concerning donor viability and the absence of protein concentration and type analysis in the exosome characterization test that still not using NGS, the result of this study proved that PRP-Exo can promote the odontogenic potential of hDPSCs, and the expression of TGF-β1 will arise on day 7, correlated with the differentiation process of hDPSCs, then DSPP expression increased on day 14 ( Fig. 5 ) as SIBLINGS' role in mineralization and crystal growth during odontoblast-like cells formation. 41 42 43 One of the critical implications of the *in vitro* findings is the potential for PRP-Exo to facilitate dentin–pulp complex regeneration. While *in vitro* studies provide promising insights, translating these findings into clinical applications is the goal.

## Conclusion

The expression of TGF-β1 and DSPP of hDPSCs after being cultured in PRP-Exo-conditioned media proves that PRP-Exo has good potential ability in inducing dentinal complex regeneration. These findings suggest a promising potential future for PRP-Exo in dentistry, especially in the regeneration of the dentin–pulp complex, offering potential solutions for tissue regeneration and oral health improvement. Further research and clinical trials will be instrumental in realizing the full therapeutic potential of PRP-Exo in dentistry.
